# Assessing the health and development of ART-conceived young adults: A study of feasibility, parent recall, and acceptability

**DOI:** 10.1186/1742-4755-5-7

**Published:** 2008-10-28

**Authors:** Jane RW Fisher, Karin Hammarberg, HW Gordon Baker, John C McBain

**Affiliations:** 1 Key Centre for Women's Health in Society, Melbourne School of Population Health, University of Melbourne, Melbourne, Victoria 3010, Australia; 2 Department of Obstetrics and Gynaecology, University of Melbourne, Melbourne, Victoria 3010, Australia; 3 Melbourne IVF and Reproductive Services, Royal Women's Hospital, Carlton, Victoria 3010, Melbourne, Australia

## Abstract

**Background:**

Assisted reproductive technologies (ART) to treat infertility have been available for nearly three decades. There have been a number of systematic comparisons of the health and development of ART-conceived with spontaneously-conceived (SC) children. Data are equivocal, some finding no differences and others that there are more health and developmental problems in the ART group. It is agreed that perinatal mortality and morbidity are worse after assisted than spontaneous conception and the impact of the hormonally altered intrauterine environment on puberty and later fertility of offspring are unknown. To date however, there has been no investigation of the health and development of ART-conceived young adults, including from the world's few prospective cohorts of ART conceived children. Obtaining these data requires contact to be made with people at least twenty years after discharge from the treating service. Given the ethical difficulties of approaching families to participate in research up to two decades after cessation of treatment, the aim of this exploratory qualitative investigation was to assess the feasibility and acceptability of approaching mothers treated for infertility prior to 1988, and their recall of the health and development of their ART-conceived young adult children.

**Methods:**

Mothers treated for infertility at the Royal Women's Hospital Reproductive Biology Unit in Melbourne, Australia prior to 1988 were approached by a senior clinician and invited to participate in individual semi-structured interviews which could include their partners and/or young adult children if they wished. Recruitment continued until theoretic saturation had been reached.

**Results:**

Ten mothers, two of their husbands and five young adults participated in interviews, and the health and development of 15 ART-conceived young adults were described. The experience of conception, pregnancy, birth and the health and development of the children were recalled vividly and in detail. Families were pleased to have been approached and supported the need for systematic data collection. Mode of conception had been disclosed from childhood to all the offspring.

**Conclusion:**

With careful and sensitive recruitment strategies it is feasible and acceptable to contact women treated for infertility at least two decades ago and their families, to assess the health and development of ART-conceived young adults.

## Background

In vitro fertilization (IVF) was developed in the late 1970's and the first child was born as a result of this technique in England in 1979. Since then a number of related techniques have been developed including Gamete Intra Fallopian Transfer (GIFT), Intracytoplasmic Sperm Injection (ICSI) and embryo cryopreservation. The term commonly used to describe all these techniques is Assisted Reproductive Technologies (ART), which are now well-established treatments for infertility. It is estimated that since IVF became available, more than one million children have been born as a result of ART [1].

The health and development of children born as a result of ART has been investigated in a number of studies of varying methodological rigor. In broad terms these can be considered in three categories. A group of studies has examined the obstetric and perinatal outcomes of pregnancies and there have been a number of investigations of the psychological, physical and cognitive development of children aged up to thirteen years. There is also a body of literature concerning general parent-child relationships, parental attitudes, parenting stress and adjustment to parenthood after ART treatment.

Perinatal mortality, morbidity, gestational age and infant birth weight are generally poorer after assisted than SC pregnancies [2-4]. These have been attributed mostly to the higher rate of multiple births after ART, but apart from the independent risks associated with multiple birth, ART singleton infants have significantly higher odds of perinatal mortality, preterm birth, low and very low birth weight and of being small for gestational age [5-9]. It is generally argued that these increased risks are due to older maternal age and the pre-existing fertility problems, but it is not possible to exclude risks related to the technologies themselves [10-13]. Adverse neonatal outcomes might have lasting developmental and health consequences and a recent systematic review of 25 studies found a 30 – 40% increased risk of birth defects in ART children [14].

Overall however, comparisons of ART and SC children have found few differences in cognitive, social and emotional development between these groups [15,16]. Only a small number of investigations have focused on physical health and development and most included only young ART children, aged between 18 months and 9 years. Of the studies that included older children, one compared quality of parent-child relationships in families with ART, adopted and SC children aged 12 and found few differences between the groups [17]. Another compared malformation rate, height, weight and school performance between ART children aged 6 to 13 with the general population of children of the same age and found no differences [18]. Leunens et al. [19] compared 109 ten-year-old ICSI-conceived singletons born after at least 32 weeks gestation with 90 SC singletons of the same age and found that the groups were comparable on measures of intelligence.

In contrast, Knoester et al. [20] compared behavior, parenting stress and child's quality of life between 110 ICSI, 110 IVF and 110 SC children aged five to eight in the Netherlands. The prevalence of autistic spectrum disorders 'seemed higher' in children born after ICSI, but this observation was noted by the authors to require confirmation in larger cohorts. Otherwise there were no between group differences and most children were regarded as having normal psychosocial adjustment. Koivurova et al. [21] compared post-neonatal hospitalization rates and health service utilization between IVF and SC seven-year-old children in a community based data linkage. They found that IVF singletons had more frequent hospital admissions which were of longer duration than SC singletons. There were no between-group differences in these rates for twins, but multiple birth is more common after ART. Most of the hospital admissions were for common childhood gastrointestinal and respiratory infections. Although the study was not of adequate size to test for the significance of between group differences in rates of disorders affecting the central nervous system, the authors reported that seizures and cerebral palsy occurred at double the rate in IVF than SC singletons and that this rate was even higher for the IVF twins.

There is also an extensive body of work studying fetal programming and intrauterine exposures that may influence later life physical and reproductive health [22-24]. Very low birth weight appears to be linked to increased risk of a range of health problems in later life and intrauterine growth retardation may be associated with premature puberty and polycystic ovary syndrome (PCOS) [24-26]. Tentative evidence suggests that there may be a higher prevalence of some very rare imprinting disorders such as Beckwith-Wiedemann syndrome (BWS) after assisted than spontaneous conception [27].

There is uncertainty about the potential effects of the intrauterine hormonal environment on the later development of the reproductive system of ART children. In an investigation of seven ART infants between the ages of five and twenty-one months investigated for signs of precocious puberty at the New York University School of Medicine, it was concluded that these changes might reflect the altered intrauterine hormonal milieu after ART treatment. The authors recommended that IVF children should be monitored throughout childhood and further into adolescence and adulthood to determine onset of puberty and later fertility [28].

To date, we have been unable to find any studies, which have examined the health and development of ART-conceived adolescents and young adults, including from the world's very few prospective cohorts. Despite the evidence about the health and development of children, it is still not known whether the apparently less favorable perinatal outcomes observed in ART children influence physical health and development through puberty and into young adulthood. The collection of comprehensive data in a systematic investigation of ART-conceived young people requires a cohort to be established from people who were discharged from treating services up to eighteen years ago. In order to undertake this, it is essential to establish whether their parents will be accessible; recall the events of conception, pregnancy, birth, health and development accurately; find it acceptable to participate in an investigation of this kind; have discussed mode of conception with their children or agree to the young people being invited to participate.

To inform future systematic surveys, the first aim of this exploratory study was therefore to establish: the feasibility and acceptability of approaching mothers of IVF-conceived young adults; parental recall of the health and development of their IVF-conceived child; and whether mode of conception had been disclosed to the child. The second aim was to ascertain the matters that these families regarded as salient and important for inclusion in a future survey of the health and development of ART-conceived young people.

## Methods

### Setting

The third child in the world and the first to be born in Australia followed successful IVF at the Royal Women's Hospital (RWH) in Melbourne in 1980. As one of the pioneer IVF programs the RWH Reproductive Biology Unit (RBU) treated large numbers of women with comparatively high pregnancy and live birth rates.

### Ethics

Women treated for infertility at RWH-RBU more than 18 years ago would not expect to be contacted by the service or to be invited to participate in research related to their child's conception. As this had the potential to be distressing, ethical approval was given for a personal introduction to the study to be made to women who had given birth following infertility treatment at RWH-RBU prior to 1988 by a member of the original treating team. Those who agreed were asked to give permission for a researcher to contact them. Approval to conduct the study was given by the Royal Women's Hospital Human Research and Ethics Committees and the University of Melbourne's Human Research Ethics Committee.

### Sample and recruitment

Almost 200 children conceived with IVF at RWH-RBU were born by December 1987 and at the time of this study were all aged at least 18 years. As the mothers and not the children were patients of the service, only they could be approached to participate in this study. The RBU database of names and addresses was checked against telephone directories and those for whom a current address could be identified were included on the potential participant list. Permission was given by the Human Research Ethics Committee for J McB a senior clinician who had been a member of the treating team at this service since 1980 to telephone women on this list to describe the study and ascertain their willingness to be contacted by the researchers. The list was alphabetical by surname and in order to minimize selection biases, telephone calls were made consecutively from the beginning of the list. Recruitment continued until theoretical saturation had been reached.

### Procedure

Women who agreed to be contacted by the researchers were sent an information package and a consent form which they were asked to sign and return if they agreed to be interviewed. When the consent form was returned telephone contact was made to make an appointment for the interview. At this point the women were invited to bring their partners and/or young adult children to the interview if they wished to. Family members who attended were asked to sign a separate consent form when they arrived for the interview. Members of the same family could elect to be interviewed separately or together.

We chose semi-structured interviews as the most appropriate method of investigating a complex and previously unexplored field of experience. A narrative approach in which participants were invited to recount their experience of infertility, assisted conception, pregnancy, childbirth and the health and development of their children to young adulthood was used. Techniques of open ended questions were used to elucidate unelaborated elements of the experience and specific questions were used to establish matters of fact including dates and the young person's developmental stages. At the end of each narrative if it had not already been described, we asked specifically about: disclosure or non-disclosure of the mode of conception; the perceived need for a survey of the health and development of young IVF-conceived adults; what aspects of health and development should be covered in a future survey and how contact could best be made with parents of IVF-conceived young adults.

The interviews were conducted at a place and time that was convenient to participants. These included a private room at the Key Center for Women's Health in Society, an academic Centre at the University of Melbourne or their homes. All interviews were conducted by JF and KH who are clinicians experienced in the psychological aspects of reproductive health. The interviews were tape-recorded and transcribed using coded identifiers.

After multiple readings of the transcripts by the researchers, themes were identified into which quotations from participant narratives were grouped. Those reported here were selected to illustrate the range of responses, with individuals identified by pseudonyms.

## Results

### Feasibility of contacting mothers of ART-conceived young adults

Approximately 50% of the addresses in the medical records were the same as those listed in a current telephone directory. All of the 13 women contacted by JMcB were willing to receive written information from the researchers. Of these 10 signed and returned the consent form and were interviewed.

In all, ten mothers of IVF-conceived young adults, two of their male partners, four IVF-conceived young adults and one spontaneously-conceived sibling participated in interviews. All elected to be interviewed as family groups. Together the health and development of fifteen IVF-conceived young adults was described and these included: six singletons, six individuals from sets of twins and three who were triplets. All these young people were conceived before ICSI was available as a treatment for male infertility.

All participants provided detailed spontaneous responses to the questions and the interviews lasted one to one and a half hours.

### Maternal recall of conception, pregnancy and birth, and early parenthood

#### Conception

Participants described reproductive histories characterized by a slow realization that difficulties in conceiving were not going to resolve spontaneously, that the cause of infertility was not always clear and that gaining access to treatment at that time was exceptionally difficult and beyond usual experience:

"I thought I would be able to have children three years after my son was born when I was 21. Just didn't eventuate.... I had an ovarian cyst and then they said I had endometriosis and I tried all medication and 13 years later..., you see it wasn't recognized that you could go on the program because you had endometriosis. I had all these laparoscopies done, I've lost count, and so I would go on the famous Clomid, and then I went on to Dr E, he was a fertility specialist and went through all the injections and what have you......, nothing." *(Mary was mother of a son conceived spontaneously when she was aged 21, and twin daughters, conceived after one cycle of IVF in which four embryos had been transferred, thirteen years later)*

"Initially when we first went into the pre-IVF there hadn't been any successes whatsoever, 1977/1978 I think...and you really had to have criteria of having had a live birth prior to being accepted ... and you had to have everything checked out and why you weren't conceiving and they found that my Fallopian tubes were badly damaged." *(Genevieve had given birth to a son at the age of 16 and 18 years later, after six treatment cycles in a five year period, an IVF-conceived daughter)*

"Well, we got married young, I wasn't quite 20 and I think when I was about 21 we thought we should have a baby. And it just didn't happen. And I went to my local GP and he did some tests with me and told me that he couldn't work things out and that he would have to refer me on to Melbourne and I said did L [her husband] need to come. Now, we were two country kids, I'm 21 years old and L is three years older and we're pretty naïve." *(Lynette experienced 13 treatments for insemination during natural cycles; followed by ovulation induction and two cycles of IVF, all using donated sperm to conceive her only child, a daughter)*

"...I mean I had been trying for a long time prior to that and the endometriosis was picked up at one stage, like a couple of years..... but I had none of the symptoms of endometriosis, I didn't know I had it, I didn't have any of the pain or whatever happens to a lot of people with it. And then I was on medication for quite a long time just to clear it up .... but prior to that, prior to the treatment for infertility and looking into things, there was never any real reason, like there was nothing major, so perhaps a little bit of this and a little bit of that and it all just came together to make lack of fertility." *(Katherine conceived her son after one cycle of IVF and gave birth to a spontaneously-conceived daughter two years later)*

The investigation of fertility and the diagnosis of infertility were recalled by some as experiences of shame, which appeared to have persisted:

"I can't tell you how bad I felt about myself. It was like a social leprosy when I realised. We were married for 11 years before I had the girls and we had 8 years on Clomid. First of all trying to get someone to even believe that you can't fall pregnant was a major thing. ...they had to ask you very personal details..., you know, does your husband know where to put his penis, like hello, and it's really embarrassing and degrading...". *(Wendy had IVF twin daughters, several subsequent miscarriages, an ectopic pregnancy and then two spontaneously conceived children)*.

"They weren't really good swimmers. It's certainly devastating to be told that you can't have children and...But you tend to get over that and go on...A counselor suggested that crying might be helpful. I didn't take too much notice of that, I would go away and I would deal with things in my own time, my own pace, work through it." *(After prolonged efforts to conceive, including through artificial insemination using his sperm, Lawrence, Lynette's husband was found to have anti-sperm antibodies. Treatment with cortisone rendered him vulnerable to infection and ultimately he agreed to the use of donor semen)*

In addition to the personal humiliation of being found to have compromised fertility the social climate was influential. At the time these informants had been seeking to conceive, infertility treatment was new and publicly contentious. Some religious denominations prohibited infertility treatment and together these contributed to a perceived need for secrecy:

"He [the doctor consulted] was at the M Hospital which was a very strict Catholic organization and we're Catholic and he said 'I'm going to recommend that you go to the Royal Women's Hospital', but he said that it would have to be kept really low key so that people didn't know because in those days IVF was very controversial." *(Wendy)*

"So we then went to adoption and that was seven years after we started trying. And then when S [their adopted son] was two we thought we would have a second child, so, we went through the artificial insemination. ... prior to that we had tried to adopt a second child and we were not able to. We went to the Catholic Welfare Bureau and they just didn't consider us 'good parents'. They had their reasons.... I hadn't 'resolved my infertility problem', whatever that means. And they felt that there should be no treatment whatsoever." *(Margaret, whose infertility was unexplained, was treated with ovulation induction and then had an IVF-conceived son, followed 16 months later by a spontaneously conceived son)*

Treatment had involved great intrusion and social disruption. Participants recalled vividly and recounted in minute detail the technical procedures; hospital admissions of several days after embryo transfer; need for repeated general anesthetics; decision-making about oocytes; lack of privacy; financial costs and the waiting. Many were moved to tears as these experiences were recalled:

"And the daily routine, for bloods, for urine test, scans and once you're in, you're in for about four days and wait for a period of time, and we had to have a catheter inserted that you got to walk around with .....And everything was high level of 'oh, will it happen this time?' If only I could get to this point or that point ... Yeah, that's what I'm thinking, that is still present even today...the anxiety." *(Genevieve)*

"We were all in there at 5.30 am, you could see when you got into the passage that there wasn't just two or three of you, there was three dozen of you..... You know at times you just thought I'm not going to do this, I'm not coming back next time. But J (treating clinician) was very good in as much as like 'I haven't come this far to let you go away'. So we just kept on going and the 19th time, he rang me at work and said 'Right, get on the tram now, get going straight' which was wonderful and it was the three day episode when you were there for three days and you had your urine tested every four hours and injections and blood tests every three hours and some of the girls had done this probably six times and had no good results. So, it gives you a great appreciation of how lucky I was actually." *(Nora gave birth to IVF twins: a daughter and a son after 19 stimulated cycles all but the final one of which had failed to produce harvestable ova)*

"....the news came back and three of the girls had no embryos so they would get up and go home while you are happy because you have embryos and you've got these other girls with no embryos and they were sort of packing up. Then we had the embryos implanted and then they would tip the bed up, like head down, 24 hours, and they said 'Don't move'. You weren't allowed to even brush your teeth. And the Indian lady next to me she never even, like when they brought the food around, (you had to eat your food upside down which was very difficult), she never moved a muscle, she never ate, she not even wriggled a toe. But hers never took...." *(Wendy)*

"...and in that first try I had one apparently very, very healthy egg produced and I was asked if I would donate that egg for research and then we could go in and try again. In hindsight I think I was an extremely stupid person to say yes because I thought yeah OK... it was for research. They were happy and 'you can come back in three months' and blah, blah, blah, blah, blah. Well, the three months then became 12 months and I kept ringing, 'when am I coming in, when am I coming in'. So, you're panicking, what's happening from now. And we ended up with six tries in five years. You had limitations, you saw women that you went through with that were not allowed to try anymore or this isn't working and it really really stressed me." *(Genevieve)*

#### Pregnancy and birth

Pregnancy and childbirth were also recalled in great detail, and for some informants, with intense emotion. Almost all had involved unexpected and demanding events, including: vaginal bleeding and threatened or actual fetal loss, hypertension, prolonged bed rest, antenatal hospital admission, premature labour or operative birth:

"...when we had the scan, I mean you don't know, ... when they said 'Oh look, there is one' and then 'There is another heart beat' and then 'It's two, it's two' and then they said 'There is a third heart beat' and I heard this 'clunk', on the floor, he [my husband] was gone. We ended up having twins ... I started off with two girls and a boy and then we lost the boy ....hmm, at about 12 weeks. But he stayed in, we actually didn't lose him, something else happened, he was mummified or something. And then I was in the Mercy Hospital for three months because I had really bad high blood pressure, threatening to stroke, I had toxaemia." *(Wendy)*

"...and then at 27 weeks I went in, I had a show and the cervix was open partially so he said you're here for however long. And then I got to 36 weeks, well I had PE, high blood pressure and that so at 36 weeks they did the caesarean. And I had a huge postpartum haemorrhage and everything that went with that." *(Pauline, adopted a son seven years after trying to conceive. Her husband was treated with surgery to repair a hydrocele and 'injections' to improve sperm motility. She was treated for endometriosis before conceiving and giving birth to triplets: a daughter and two sons after IVF treatment and the transfer of four embryos)*

"Well, I started bleeding probably about week nine and after that I spent 26 weeks in the Women's Hospital. I came home a few weeks and then I ended up going in again. It was a very, very nervous time. The whole time was a nervous time, it didn't matter if it was week 16 or week 25, you just never knew. And because I was in there, unfortunately you do actually see how many people lose babies. And that in itself is horrific really because you realize, like every day you can hang on is another day ..." *(Nora)*

"When we came down, we had to come back for the six week ultrasound, because we had four embryos implanted, that was before they cut down on how many you could have put back, there were two heart beats. It was very exciting. ...but I obviously naturally aborted one without upsetting Miss Muffet [her young adult daughter who was present for the interview] here! So when I went for my 14 week scan at home [in the rural area where she lived] I really thought I was having twins and I remember the radiologist, because I work at the hospital and all the girls [her co-workers] would come to the scan, and he was going 'Well there is only one baby here. Who told you there were two babies?' Well, that really...the girls just disappeared and I can remember, I just had to take myself off...but anyway we got one beautiful one who arrived three weeks early but fit and well ..." *(Lynette)*

"I would have said it was difficult [the pregnancy]. ... It was probably 18 weeks when we had two ultrasounds and I had a bit of bleeding, it was on the point of whether the pregnancy would hold on or not." *(Ruth, gave birth to an IVF daughter on the 13^th ^embryo transfer and two years later, an IVF son)*

One participant's pregnancy proceeded to term, and culminated in the induced vaginal birth of baby of average weight, however, it was recalled as having been extremely alarming:

"...so the whole pregnancy was horrendous. You would come in for your scans you would come in for your checkups. You're obsessed. You are obsessed with that conception, absolutely obsessed. And, all the way through it, you'd come in, you'd have your check-up you'd go home happy, but you would be less than happy within 24 hours, wondering if you were still pregnant.... It should have been a lovely, lovely experience but I worried all the way, every step of the way. Every step you thought, and of course you are reading and taking information from everywhere, and each trimester, like this can happen and that can happen and you think oh, 'The baby is not moving, why isn't the baby moving, am I still pregnant?' Or you would have movements and you would think 'there is not much movement, why not, is the baby all right?'." *(Genevieve)*

#### Early parenthood

Given the number of multiple and premature births these participants experienced, it is not surprising that the early months of motherhood were recalled by some informants as having been overwhelmingly demanding and difficult. Again, these were described in vivid detail and with great emotional intensity:

"The girls stayed in hospital for another 4, 5 weeks after that. I had massive blood transfusions and it was before AIDS. The blood got cleaned in March or February of 85, and this was October 84. I mean they were pumping it in one end and it was coming out the other. Mr T [the treating obstetrician] said to me that he had quite a lot of ladies from Sydney that had been diagnosed with HIV and passing it on to their children and he recommended that I did not breastfeed, so I did not, which is a big sacrifice...". *(Wendy)*

"One of them was growth retarded, the other two were fine... they were reasonable weights. They all got jaundice and I wasn't able to breastfeed, I didn't have enough milk. And we took home T at three weeks and then the other two came the week after. While M was at the Grey Sisters [a Melbourne residential early parenting service], T had inguinal hernia, so he was at the Children's [The Royal Children's Hospital] and I was still bleeding and then after that T came home, they were all home then and it was just horrible. The first six months were very, very labour intensive, if that's the word you use. It was just feeding, you know, winding, changing... and then C got pneumonia at three months, she was at the Children's again. T had something... we were in and out of the Children's for the first six to eight months and then after that they were all fine." *(Pauline)*

"So, a month later, he [her husband] goes back to work but you are then still holding the baby that's still constantly crying. And the only way she is happy is if you keep on feeding her and cuddling her. We also had these high expectations, you want to do the right thing by your baby, no formula feeding, must be breast milk, no dummies, don't give your baby a dummy, it's bad for them. So you go through the no dummies. And you're a mummy dummy." *(Genevieve)*

Other informants described early adjustment to motherhood as having been more straightforward and pleasurable, most identifying infant behaviour and manageability as a salient determinant:

"They were very good babies. I must admit, they were no problem at all. They fed well, we came home and they were both basically four-hour feeders and sleepers, the only difficulty naturally enough is that you've got two babies so when you finish with one you've got to start with the other one. Really, no problems with them whatsoever." *(Nora)*

"Yes, normal weight, normal everything. He was a very healthy little boy.... and he was an easy baby, he was a lovely little boy and no trouble, very settled, just gorgeous..." *(Katherine)*

"Oh, she cried a bit initially, she had a bit of colic, but she was a reasonably good baby." *(Ruth)*

The psychological legacy of infertility, its treatment, complex pregnancies and demanding early adjustment experiences appeared to be substantial in that four of the ten mothers reported that they had required treatment including hospital admission for mood disturbance in the first postpartum year:

"Hmmm, but then I got postnatal depression, ... but the ladies in the South wing, at the M [hospital where she had given birth] was the first hospital to recognize that it wasn't an insanity thing." *(Wendy)*

"It's probably four or five months before the curtain fell on mummy she landed in hospital for a while. ... I eventually went to the GP and the GP put you into a private hospital, a local private hospital ... and assigned to a psychiatrist... but the hospital wasn't set up for mothers and babies. It was just a normal room and you had the baby with you. So, not a good experience, took a long time to get over. And everything was, you know, she would cry, why is she crying, has she got cold, has she got this has she got that? What's wrong, something must be wrong." *(Genevieve)*

"I actually had postnatal depression after she was born." *(Lynette)*

"...so they came home and that was pretty horrendous, to say the least! M, the growth-retarded one had always been a bad feeder, he was a horrible, horrible child who screamed the whole time, so he went into the Grey Sisters' for a week and they fixed him up..." *(Pauline)*

### Maternal recall of the health and development of ART-conceived young adult

The early infancy, childhood and adolescence of the index young people were also recalled and described in great detail. Parents recalled both a general impression of overall developmental progress and were asked about some specific aspects of health and development.

#### General developmental progress

"Developmentally, to me they have all had normal milestones. ... M was always behind in his feeding and he was always a bit smaller ... I think they all crawled similar time but he took a lot longer, he didn't walk until he was 15 months. He could stand and everything, there was nothing wrong with him... He was a very bright child, I knew right from the beginning that he was bright, which proved to be true, very true." *(Pauline)*

"I guess he was just like any other, just like my other son. He walked at about 13, 14 months I think, he was a little bit colicky to start with, went to school at five, he achieved really well." *(Margaret)*

"And the first day at school ... I took them into the classroom, they sat in the circle and they didn't even wave goodbye. They were just so happy to be trying and really had no problems through school. I think they were quite well adjusted and moved into growing up. " *(Nora)*

"Walked early, very good in her athletics, very good with swimming, very good with netball, basketball. Very spoilt, yeah, very spoilt." *(Marion, mother of a spontaneously conceived daughter and eleven years later, after 'at least four' embryo transfers, an IVF daughter)*

"He was a very healthy little boy with all the developmental things. Once as a little baby he was in hospital overnight for vomiting, but he's had no other problems at all, he's had no ill health or issues; he was just a normal little boy right through primary and secondary school." *(Katherine)*

#### Puberty

They were also asked more specifically about age of puberty, including menarche, which were recalled clearly, often with confirming details:

"Yeah, my girls were really late. S got her period at 15 and E at 16. So, I remember it well because it wasn't that long ago." *(Wendy)*

"Grade 6 I think, I think S was first, she had her periods in grade 6 and L I think a few months later." *(Mary)*

"C came a bit earlier, I think girls often do that... they were all quite athletic and lean and you know, but she went to secondary school and put down some fat, got her period and stopped growing. So she is only little, she is only 5 foot nothing, she was 13 when she got her period. ... the boys, they were probably 12 months later. T tended to be a bit behind the others...and he was the biggest baby which is strange." *(Pauline)*

"Oh, I think much the same [as other boys], like I didn't notice any difference " *(Margaret)*

"Well, D was probably a little bit of a late developer; she was probably almost 15 before she had any breast development or had a cycle. But after that she certainly, she's a big girl now.... and cycle wise, apart from having painful periods...like she was regular once she actually started so no problems in that department." *(Nora)*

"Puberty was earlier actually ... it was early secondary school. Within the range but I remember looking at photos, he kind of was very tall, he was the tallest one in primary school but then kind of stopped. I looked at him with some of his friends and yeah, puberty did kick in early." *(Katherine)*

#### Progress at school

Participants were asked specifically about the young adult's overall intellectual development, physical abilities, participation in social activities and progress towards an occupational identity:

"Not overly fine, but good, they studied. Like my girls out of the 10 for effort they always did 10, but they might have only had an ability of 7. But they always did better because they were working hard. And I mean their ENTER scores [Australian final secondary school examination result ranking], E's was 84 point something and S's was an 82. S is extremely creative, artistic, mega hard worker, likes hard work. E is a wonderful sports woman, mega hard worker and highly intelligent in the commerce/maths side." *(Wendy)*

"It's their last year of Uni. C did journalism and ....T did an Arts degree and business and he hopes to get a job soon, and M did behavioural science and he is going back to do honours...." *(Pauline)*

"...played a lot of sport as he still does. Secondary school, I always thought he was I guess maybe the plodder of the two, not quite as bright as his younger brother. He's just got, oh, 12 months ago, his university degree as a civil engineer with honours! So, what more can I ask for? And he is a really gentle, caring, lovely boy." *(Margaret)*

"I think G was probably pushed into school too soon, she moved in and she struggled. All the way through primary school she struggled with the first half of the year, did much better in the second half. Did a lot better in secondary school. Socially probably because she didn't have friends around as the area we were in still didn't have children in her age bracket and because we didn't move out of that area she seldom had friends, seldom had any kids to play with or integrate with." *(Genevieve)*

Yes, well, we did get a little bit concerned with A [his IVFC son]. We did get a little bit of help from a speech therapist too at primary school. So, there may have been a little bit of...it might not have been exactly the natural track there for a while [regarding the children attending a small local primary school]. Well, it helped A especially because he'd get, he could get one-to-one help. Whereas had it been in a big primary school, he could have got lost." *(Roger, Ruth's husband and the father of their IVF daughter and son)*.

"School was fine, he did well. He finished his degree two years ago. He went straight from year 12 and did a Commerce degree. And then he went straight from finishing the degree to travel in South America, and now he is in France so you can't see him!" *(Katherine)*

"She was Dux of the school and school captain. Mum and Dad were pretty proud...and the fact that we didn't go to university but S got in." *(Lynette)*

"She always got good reports at school." (*Marion*, *referring to her daughter who is now working as a registered nurse)*

#### Major illness and hospital admissions

Informants were asked explicitly whether there has been any major childhood illness, including whether hospital admissions had been required. In general it appeared that the episodes and illnesses described were within expected patterns for young people:

"E had epilepsy, but it was not caused... it was caused by two big impacts on her head. Like when she was in grade one she was in a roller skating party when bang on the back of her head. They didn't call me until I came to pick her up and she had the size of an egg at the back of her head. And then, like a month later she was jumping off our bed and we've got a wooden bed and she slipped off and hit her head again. And so she was on Epilim until she was grade six and then they weaned her off and she hasn't had anything since so it was more like an impact. And S has just been diagnosed with polycystic ovaries." *(Wendy)*

"She had her tonsils out at four. She had a bad virus a couple of years ago that wiped out her immune system, but I think she is getting over that. She had asthma early, but just childhood asthma that she grew out of. So, I really couldn't say that there was anything different health wise between the two." *(Marion, comparing her SC and IVF daughters)*

"There were no hospital admissions or illnesses outside the sort of childhood illnesses. ....a bit of a knee problem but that was later on and that was sport related." *(Genevieve)*

### Parental disclosure of mode of conception

One of the risks of conducting a survey of the health and development of ART-conceived young people is that mode of conception might not have been disclosed to them. Specific enquiry was made into whether mode of conception had been disclosed to the young person and how this had taken place. In this group, all young people had been informed about mode of conception, for most, from early in life:

"Right from the beginning, just like with S that he was adopted. There was an article in the paper by F [a local conservative ethicist] saying that IVF children weren't loved you know.... And one of them got straight onto the e-mail and said you know, if we were unloved I don't know [about it]." *(Pauline)*

"When he was young, when you know people would tell how babies were conceived we would say you were a little different, yeah, he's always known." *(Margaret)*

"Mum and dad were very open and I don't know how I computed it at that early age but I still was able to understand that there was mum's eggs, dad's sperm and all that sort of stuff and I remember in primary school probably about grade one or grade two trying to explain to people, because I thought oh, this is something to talk about, pretty cool." *(Grace, who is daughter of Genevieve)*

"We did (tell the children about their IVF conception). I can't remember how old they were. I think it might have been seven or eight, and we just told them that there were a lot of things that had to be done and so on, so they had an inkling... I then explained to them the process briefly because at that age you can't take it all in and I can remember probably only one negative thing, my J said to me 'Sometimes people make me feel like I am a little bit of a freak'. So I said 'Well, you are not a freak, you are exactly the same as everybody else, it's just the process that was different'." *(Nora)*

"She knew right from the start. You let them know that they are special! I let her know that she cost me a fortune! .... I never even kept it quiet when I was going through IVF. We did the petitions when Harradine [an Australian politician opposed to ART treatment] tried to stop the thing. So, I went to work, said to people 'Hey, sign this' ... I never hid it, I never thought it was something that oh, I have to hide. But having said that, there is a lot of people that I went through with, that their family didn't even know that they were going in for it. I'm from a Catholic background and they've got nothing to do with it. ...Even now, like all my friends at work know that S is an IVF baby... She is my special... she is my IVF baby." *(Marion)*

The one partial exception was that one young person who had been conceived with donated sperm had known about her IVF-conception, but only been told about the use of donor gametes when she turned 21.

"That was a lot harder... [to disclose the use of donor sperm]...I didn't really think about it too much. It probably would have been a little while sooner but I had it fixed in my mind that this is the way it was, we were to more or less sit on it and that was it. But yeah, when we did speak about it and let S know she was a donor then it has become easier. I was probably a bit worried that maybe I would have lost her.... It was a big worry..."*(Lawrence)*

"Being like an IVF child? I don't know, I've always been pretty proud of the fact, it's always been my big thing, like I tell people I was manufactured ...specially ordered. But no, Mum and Dad have always been really honest about that side of things and they have kept me articles and that kind of thing. I wondered why Mum got them all out again actually for my 21st birthday. Oh, well I found out that I was the result of donor sperm which came as a bit of a shock but it sort of didn't. I think on some level I have always known. Just because Mum and Dad were always really honest but there was something they kind of skirted through a little bit, like you'd never really got the full story. And for quite a while I used to think that I was adopted...I mean probably it would have been nicer to maybe have known that a little bit earlier as well, like to have gone hand in hand but I suppose hindsight is a good thing and they, like you guys did what you thought was right at the time. ... They sat me down a couple of days after my party and, Dad came and got me out of bed, and they said 'Oh, we have to tell you something'. They took me down to our kitchen that looks out on the back yard so it was quite nice and calm and everything. And I had a thought right then they are going to tell me that I'm not theirs...But yeah, they just sat down and they cried, and I cried, and they cried some more ... and they just said that they had to have a bit more help in making me so...it's been interesting, it's been a little bit of an adjustment. But from my point of view, Dad is my Dad." *(Shelley who is Lynette and Lawrence's daughter)*

### Acceptability of a survey of the health and development of ART-conceived young adults

In order to establish if a future population-based survey would be acceptable and of interest to families with ART-conceived young adults all participants were asked about two matters. First about the acceptability of being approached to participate in a potential future survey up to 25 years after the young person's birth and second about what matters such a survey should cover. There was a near unanimous view that this would not only be acceptable it would also be of interest to these families and that these outweighed the potential intrusion of an unsolicited approach.

"I was so thrilled. Because we were so new in the old days I was just wondering if anyone have ever documented what went on, like even just for medical students that would want to go back just to see how the old fashioned way was done, how we have improved and how much more accessible it is to anyone. Like, it's wonderful." *(Wendy)*

"I think so, yeah... I don't know anyone [else] who has had IVF children. So...I think they would ...give some information." *(Margaret)*

"Well, I wouldn't object to it. I just think I can give something back for what I got." *(Margaret)*

"Well, to be really honest, I think if you had children my age you'd probably be really glad of the follow-up, to know that you weren't sort of lost in the system and that there was still an interest. And also for the welfare of your children, to know if something was picked up, that you would find out." *(Nora)*

"I don't know, I think certainly a few of them would be like myself and they wouldn't want to say anything to anyone. Then again there would be others that would express their thoughts and would do it pretty easily but my own point of view is like I said, I would be pretty closed. [Asked if he would have filled out a survey if he had received one]... I would probably go and sit and fill it out, especially now that we have told S. I would certainly go and fill a survey out now whereas before I wouldn't." *(Lawrence)*

"I think it's a terrific idea." *(Ruth)*

[Asked if a survey would arouse negative reactions] "No, not at all." [Would young adults like her son mind being approached by researchers] "I don't think so, I don't think he would. If he was around ...he would be fine." [Would a survey be appreciated by families with IVF conceived adolescents) "Yes, absolutely." *(Katherine)*

There was a more qualified view about whether young people themselves would be prepared to complete a survey:

"I don't know, I think it would depend on the young person, I don't really know how my young person would react either. ... I mean some of them like I said, S has always known but I guess some other young people might not have." *(Margaret)*

"...neither of my children would have a problem talking to you." *(Nora)*

However, the young people who participated in these interviews all thought that a survey would be of interest to them and that it would be acceptable to be approached to participate:

"Well, I think a survey is a good idea. I suppose, I don't know how to word it but... I suppose if somebody didn't know and they had never been told and they opened it and something like hmmm, IVF. Not that that would be an issue but I suppose if your parents had never told you...." *(Shelley)*

".....I mean the thing is, like it was something for me to fill out or for Mum, working within a health care profession, you know how important research is." *(Susan, Marion's IVF daughter)*

".....Yeah, that would be fine." *(Janine, Ruth and Roger's IVFC daughter)*

Apart from the matters that had been addressed in the interview, there were relatively few suggestions of further areas for inclusion in the survey:

"Just you know what their developmental and physical health has been like." *(Pauline)*

"Whether they had maybe any physical defects ...eyesight .... speech ...illnesses, like did they have a lot of childhood illnesses ... I don't know ... Maybe growth, whether they developed in the same way as their peers..." *(Margaret)*

"I think knowing how other families react or have reacted to it [IVF] would be nice, and even how many of them are only children themselves. Yeah, and how the family gone about it because I don't know if it's IVF or if it's just how Mum and Dad raised me but we are pretty close family. So yeah, I don't know how other family units kind of operate." *(Shelley)*

## Discussion

There are some differences in the health and development of children conceived with ART compared to those conceived spontaneously, but as yet there are no investigations of these in young adults. This investigation was conducted to establish whether: it would be feasible to contact mothers treated for infertility at least 18 years ago; the conception, pregnancy, birth, the health and development of the ART young adult could be recalled and they would find it acceptable to be surveyed.

### Feasibility

This exploratory investigation demonstrates that it was feasible to identify and approach women to participate in a follow-up investigation up to 25 years after ceasing an association with the infertility treatment service. The strategy of a first approach being made by a senior clinician, who had been associated with the service since its establishment, appeared to be crucial.

Further, while the linkage of the database of names and addresses with telephone directories is time-consuming, it is feasible, with current details being found for approximately 50% of all past RWH-RBU patients. In addition to telephone directories using electoral rolls to ascertain current contact details for past patients increases the proportion that can be traced. Venn et al. [29] traced 87% of past health care users several decades after they had been treated using electoral rolls in addition to telephone listings.

### Maternal recall of events

The limitation of all retrospective data collection is recall bias. It appears however, that these participants recalled the experiences of diagnostic confirmation of infertility, fertility treatments, pregnancy and childbirth in vivid and persuasive detail. There is evidence that women recall childbirth with near perfect accuracy up to twenty years later [30]. It appears from these data, that other reproductive health events, perhaps in this case because of their high emotional salience, are also recalled in detail. In Victoria, Australia there are two infertility treatment services, each providing care for about half the population; however, there are more than sixty obstetric hospital facilities in the state. After assisted conception women are discharged from the infertility treatment service and provided with mainstream antenatal and intrapartum care in either state-supported public hospitals or by independent obstetricians in private clinical practice. Thus, while the medical records of infertility treatment could be retrieved it is unlikely that all the medical records describing pregnancy and childbirth care would be obtainable and these data are likely to have to be gathered directly from informants. These details are crucial to being able to evaluate the impact of intrauterine exposures, intrapartum events and neonatal and infant health on later development.

### Disclosure

One of the potential barriers to a survey of the health and development of ART-conceived young people is that they might not be aware of their mode of conception. It is known that this is especially likely if donated gametes have been used [31]. A matter elucidated in this investigation is that mode of conception had been disclosed to all these young people, usually from early in their lives. There was variation in how families had approached this matter and the degree of comfort that the parents and the young people felt in discussing it within the family or with their extended families or wider social networks, but in none had it been kept secret. The only exception was that while mode of conception had been disclosed, one young person had only been told of the use of donor gametes in her conception when she reached adulthood.

### Acceptability

All of the participants indicated that a full survey of all women who had been treated up to 1988 and their children should proceed; that there would be general interest in participation and that summary data about the health and development of ART-conceived young adults was of interest to them.

### Implications for future research

We conclude that it is both feasible and acceptable to approach women and their ART-conceived young adult children to invite them to participate in surveys of the health and development of ART-conceived young adults.

## Competing interests

The authors declare that they have no competing interests.

## Authors' contributions

JRWF participated in the design of the study, conducted the interviews, identified content themes and drafted the manuscript. KH participated in the design of the project, conducted and transcribed the interviews, identified content themes and helped draft the manuscript. GHWB participated in the design of the project and identified potential participants from databases. JCMcB participated in the design of the study and made the initial telephone contact with potential participants. All authors read and approved the final manuscript
